# Fatigue behavior of three thin CAD/CAM all-ceramic crown materials

**DOI:** 10.1080/23335432.2024.2303121

**Published:** 2024-01-11

**Authors:** Khaled Bataineh, Mohammad Al Janaideh

**Affiliations:** Department of Mechanical Engineering, Jordan University of Science and Technology, Irbid, Jordan

**Keywords:** CAD/CAM ceramics, dental crowns, fatigue, finite element method, minimal invasive

## Abstract

The purpose of this study was to study the effect of crown thickness on the fatigue life of CAD/CAM ceramic materials. CAD/CAM ceramic materials for the crown were virtually designed with three thickness designs of (a) ultra-thin occlusal crown average 0.7 mm thick (group A), (b) thin occlusal crown 1.1 mm average thick (group B), (c) thick occlusal crown 1.5 mm thick. The materials are: zirconia Cercon ZC and IPS e.max CAD (LD). Finite Element Analysis (FEA) simulations were carried out to estimate the fatigue lives of restorative materials. The lives for groups B and C under fatigue load were not significantly different from each other for Zirconia. The predicted lives for group A zirconia crowns, under fatigue load 50 N, 100 N, 120 N is 24 years, 4.3 years, 1.9 years, respectively. Results for crowns made of LD can be summarized as follows: under load 50 N, all groups have survived longer than 5 respectively, while under the load of 100 N, only group C survived longer than 5 years. 0.7 mm thick full contour Zirconia crowns possessed adequate endurance strength to survive under physiologic conditions. On the other hand, the crown made of LD should have at least 1.5 mm thickness to survive longer than 5 years.

## 1. Introduction

Restoration is the treatment for replacing tooth structure loss. The properties of an ideal restorative crown material are: high strength, high fatigue resistance, biocompatible, aesthetics, and color stability. All-ceramic restorations satisfy most of the previous criteria (Yilmaz et al. 2007). It has been reported that most ceramic restorations have high clinical failure rates especially if they are placed on posterior teeth (Chen et al. 2014). In order to overcome this failure problem, several restorative materials have been developed recently. CAD/CAM ceramics are developed offering extra strength and more structurally homogenous, improving the retention and longevity of the restorations.

Among CAD/CAM ceramics, Zirconia is an attractive choice for restorations due to its aesthetic appeal, biocompatibility, and high flexural strength (Kosmac et al. 1999; Tan and Dunne 2004). The flexural strength of zirconia ranges from 650 to 1200 MPa (Denry and Kelly 2008), and its shear strength is comparable to that of metal ceramics (Al-Dohan et al. 2004). Lithium disilicate (LD), a widely used modern CAD/CAM all-ceramic material, has superior aesthetic properties and color stability (Duan and Griggs 2015). On the other hand, LD is still not strong enough for posterior teeth and due to its brittleness, it is susceptible to fracture (Carvalho et al. 2014). The fracture resistance of zirconia crowns under a variety of conditions has been studied previously (Alhasanyah et al. 2013). It was reported that core/veneer thickness ratios have a significant effect on the hardness, fracture toughness, and residual stress in zirconia crowns (Millen et al. 2012).

The success and survival of restorative depend mainly on the mechanical properties of restorative materials, and the magnitude and the type of loads, the thickness of the crown material, and the shear bond strength of the cement material. The most common mechanical failure of a restored tooth is usually due to fatigue failure. Despite this fact, large number of studies have focused on static analysis. The literature lacks accurate investigations for performing fatigue analyses for restorative material. Moreover, most have simulated occlusal load by applying a single point. The stress distribution generated due to the action of a single point differs significantly for those found under oral conditions.

General ceramic preparation guidelines require an axial and occlusal tooth reduction of about 1.5–2.0 mm to ensure the stability of the crown. However, the excessive removal of tooth structure may cause potential damage to dental pulp (Lameira et al. 2015) or reduce the stability of the remaining tooth substance. The prepared teeth may be preserved using minimal invasive techniques together with a minimal thickness of the restoration. Crown thickness is supposed to have a significant effect on the stability of the restoration (Thompson and Rekow 2004). Monolithic zirconia crowns with a chamfer width and occlusal thickness of only 0.5 mm showed sufficient strength for an application in posterior areas (Nakamura et al. 2015, 2016; Sorrentino et al. 2016). However, the stability of the restoration is further influenced by the type of cementation (Nakamura et al. 2016; Sorrentino et al. 2016). Adhesive bonding improved the fracture resistance of monolithic all-ceramic crowns compared with conventional cementation (Sun et al. 2014). A study conducted by Weigl et al. concluded that 0.5 mm thick monolithic crowns possessed sufficient strength to endure physiologic performance, regardless of the type of cementation (Weigl et al. 2018). Furthermore, they found that the fracture strength of the 0.2 mm cemented crowns was too low for clinical application (Pogoncheff and Duff 2010).

Up to date, very limited number of studies are found discussing the relation between the crown thickness and survival rate of all-ceramic restorations. Numerical simulations using Finite Element Analysis (FEA) can be effectively used to predict the fatigue life of minimum crown thickness under clinical loading conditions. It is worth mentioning that the simplified static test which loads the crown until fracture does not offer valuable clinical information. Hence, the mechanical behavior of the crowns should be evaluated under cyclical loads to simulate the actual clinical chewing cycle.

The main objective of this research was to test the fatigue resistance of thin CAD/CAM ceramic crowns. It is desired to have a minimum thickness of the crown material that has adequate fatigue resistance. Minimizing tooth reduction can be achieved by using minimum thickness for the crown materials. It has been believed that all-ceramic restorations required extensive tooth preparation. The purpose of this study is to test the hypothesis that a thin crown can withstand cyclic stresses generated from masticatory forces. The materials tested were zirconia Cercon ZC and lithium disilicate (LD). The fatigue life for the restorative materials is estimated using 3D FEA.

## 2. Material and methods

This study focused on studying the effect of thickness of CAD/CAM ceramic crowns on their fatigue resistance. FEA was used in this study. Two designs of crown thickness were virtually tested under fatigue loading. The scanned die was used to design full contoured crowns using the computer – aided design/computer -aided manufacture CAD/CAM system using (Sirona CAD/CAM Mcx5milling machine Software inlab sw16.1). The STL file of the designed crown was generated. The STL files were converted into solid models using solid modeling software (SolidWorks 2018, DS Solidworks Corp, USA). Several Boolean operations were used to assure the interfacial mesh congruence. The assembly was transferred into finite element software package ANSYS 18.1 (ANSYS, Inc., USA). Virtual designs of the full contours were made as follows:

Group A: ultra-thin crown thickness average (0.65 mm).

Group B: thin-crown thickness average (1.1 mm).

Group C: typical crown thickness average (1.5 mm)

**Figure 1.**
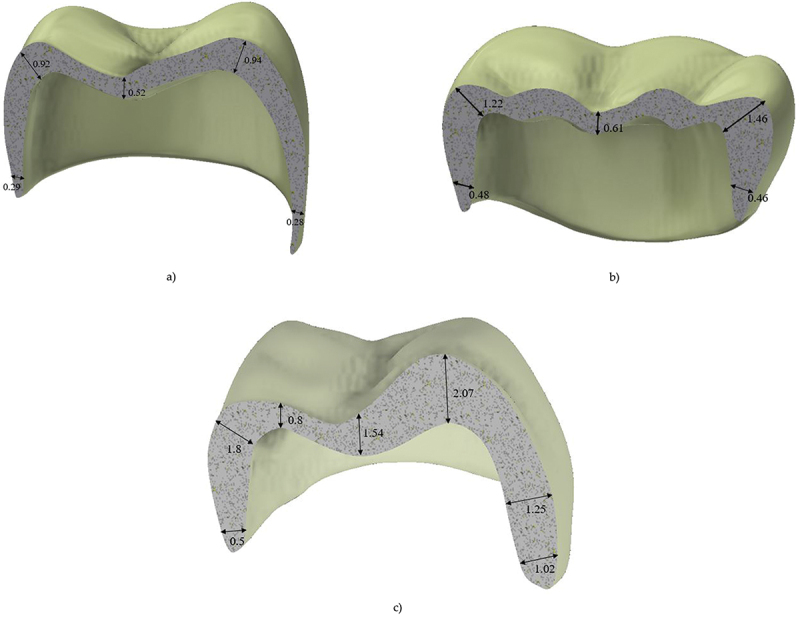
Thickness of the crown a) thin b) typical thickness crown.

**Figure 2.**
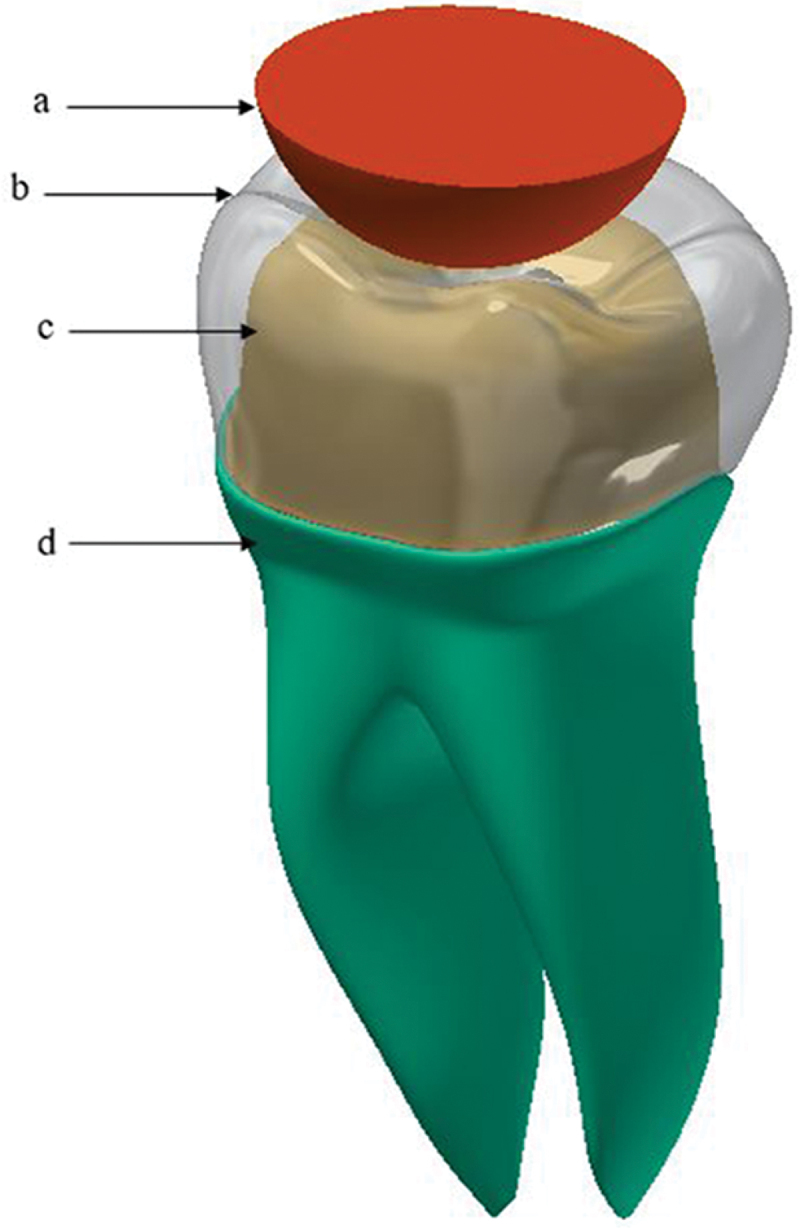
3D model of the restored first molar, (a) indenter, (b) crown, (c) dentin, (d) root.

Figure 1 shows the full contour of the three crown thicknesses studied. Figure 2 shows the 3D model of the restorative first molar assembly. The 3D model of the lower first molar crown consists of a crown, cement layers, and die (dentine). The thickness of the cement layer is 50 μm. The physical interactions between various parts of the dental restoration are modeled through bonded surface-to-surface contact features of ANSYS. Furthermore, the interface between the prepared tooth and restorations is assumed perfectly bonded.

**Figure 3.**
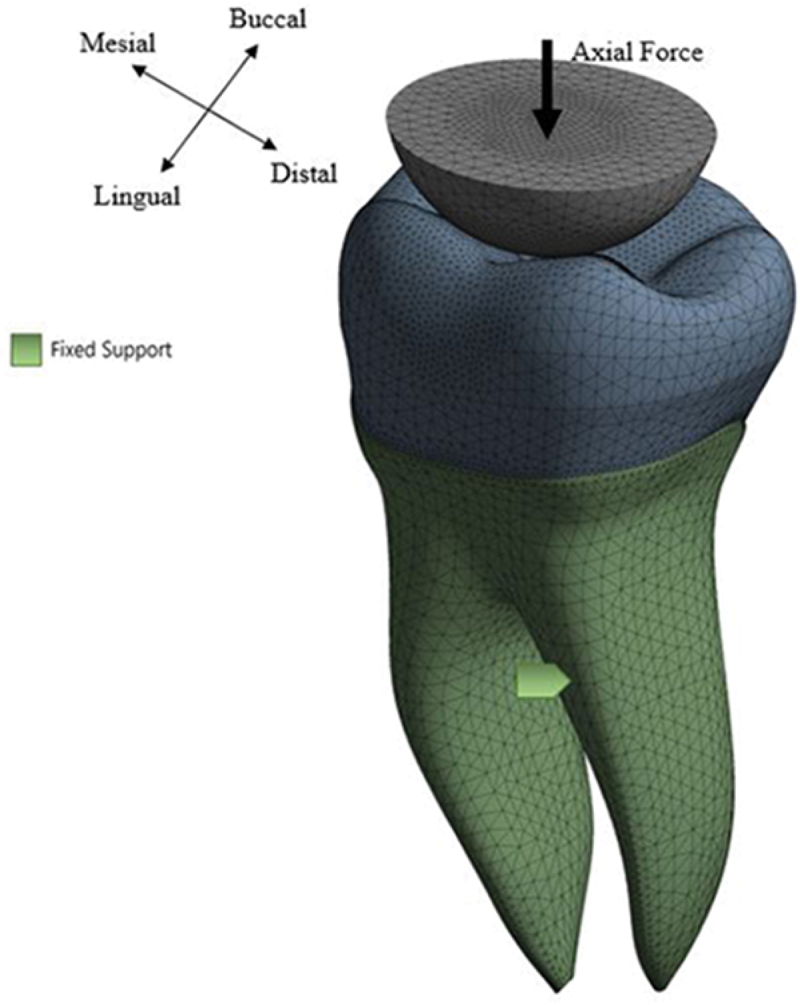
Boundary conditions and applied loading (arrow) for the used model.

The magnitude of the occlusal forces is important in determining the long-term success of restorative materials. The model was fixed at the bottom surface as shown in Figure 3. Fatigue loading was simulated through FEA by applying sinusoidal compressive cyclic indentation loading (minimum compressive load: 5 N; maximum load: **variable**). The loading was done at the center of the tooth using an 8 mm diameter ball made of a material that has similar mechanical behavior to the enamel. Each group was subjected to six values of fluctuating axial force patterns. The minimum force was fixed at 5 N, while the maximum force values were 50 N, 75 N, 100 N, 125 N, 150 N, and 175 N.

**Figure 4.**
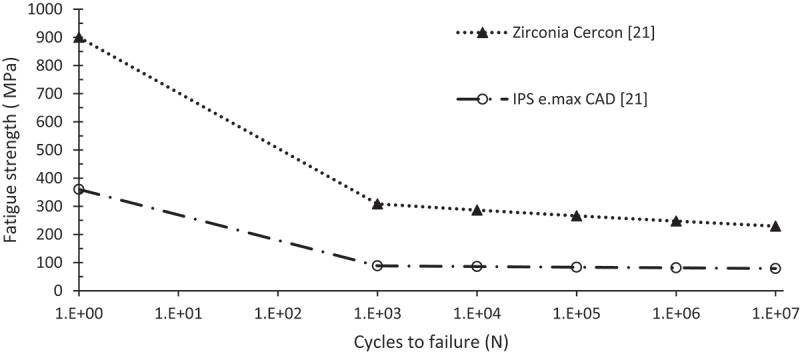
Fatigue strength S-N curve for restorative material used (Homaei et al. 2016).

The fatigue behavior of two restorative materials (zirconia Cercon ZC and lithium disilicate LD) was estimated. The S – N curve represents the stress amplitude (*σ*_*a*_) as a function of number of the cycles to failure (*N*). The mechanical behaviors of the studied materials were described by linear isotropic material models. Mechanical properties are listed in Table 1. Figure 4 demonstrates the fatigue strength versus life cycle (S-N) for the crown materials studied. The data presented in Strength – life **curves** were directly input into ANSYS.

**Table 1.** Material properties for the restored first molar model.

Material	Young’s modulus (GPa)	Poisson ratio (ν)	Yield strength (MPa)	Flexural strength (MPa)	Compressive strength (MPa)	Shear strength (MPa)	ref
Indenter (mechanical properties similar to enamel)	84.1	0.33		11.5	384	60	(Nihioka et al. 2018)
LD	95.9	0.23		356.7			(Nihioka et al. 2018)
Zirconia Cercon ZC	210	0.32		900			(Nihioka et al. 2018)
Dentin	18.6	0.31		105.5	267	12–138	(Dejak et al. 2012)
Dual cure resin cement	8	0.3				34.4	(Dejak et al. 2012)

As shown in Figure 3, finer mesh density at the occlusal region was generated due to the highly complex features of the occlusal surface. The FEM model consists of more than total 320,000 four-node tetrahedron elements was built. Frictional contact between occlusal surfaces and the indenter was simulated. Moreover, the crown was assumed perfectly bonded to the dentin. The stress distributions in the parts of the tooth were determined. Finally, the static FEA results were post-processed with fatigue stress – life (S – N) behaviors to determine the factor of safety guarding against fatigue failure and predicting the life of the restorative material.

## 3. Results

**Figure 5.**
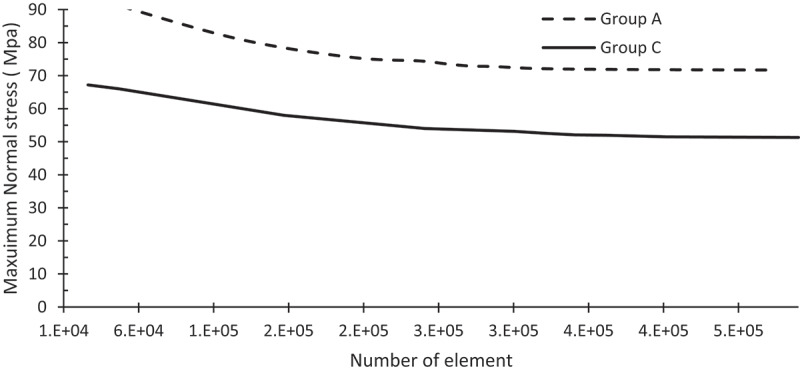
Sensitivity mesh analysis for the number of elements.

**Figure 6.**
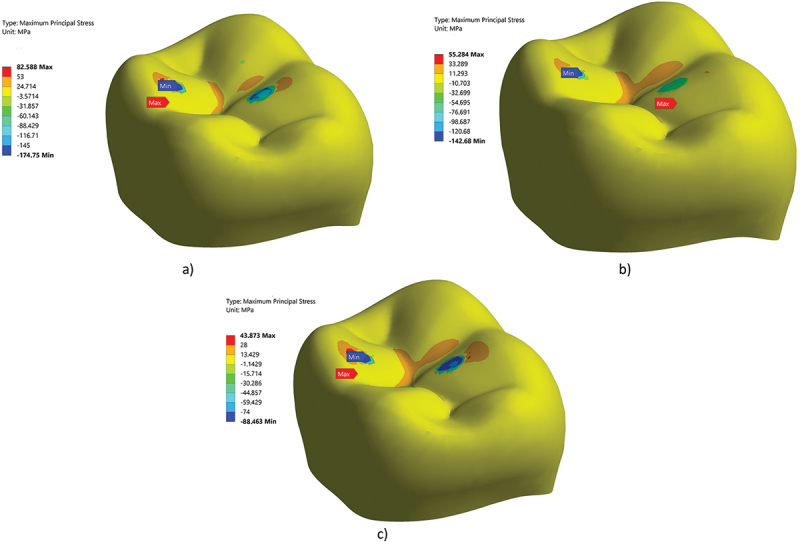
Max tensile stress for the three groups studied under axial compressive load =150 N for zirconia crown, a) group a, b) group b, c) group c.

**Figure 7.**
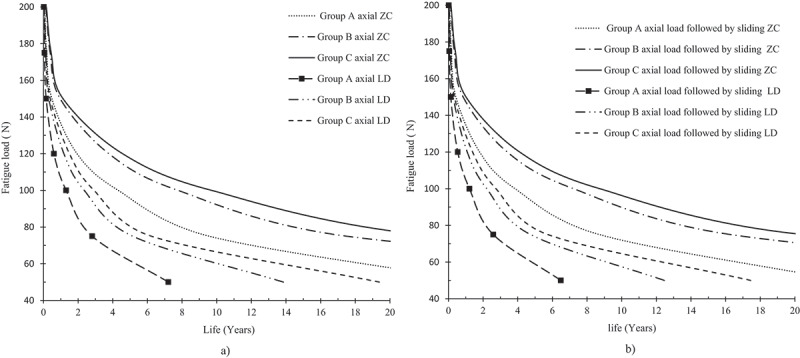
Survival rates the three groups tested under two loading conditions, a) axial loading, b) axial followed by 0.8 mm sliding.

Finite element simulations were carried out to evaluate the fatigue resistance of thin crowns. The first step was to check the mesh density of the Finite element. Figure 5 shows the mesh convergence test. The results were mesh independent at mesh density equal nearly to 375, 000 elements for group A and C. On other words, results did not change with any further increases in the number of elements. Figure 6 shows the maximum stress contour for group C under three values of axial loads. The maximum principal stress is 43.3 MPa, 55.28 MPa, and 82.55 MPa for zirconia crown under an axial load of 50 N, 125 N, 150 N, respectively. Figure 7 shows the expected lifetime in years for the crown tested in this study under two loading conditions; axial force and axial force followed by sliding motion. The life in years for group A crowns was 2.2, 1.2 under cyclic load 100 N for zirconia and LD, respectively. Adding sliding on the amount of 0.80 mm, the life reduces to 1.2, 0.5 years, respectively. The number of life in years for group B crowns made of zirconia are 24, 5.5, 2 under an axial load of 50, 100 120 N, respectively. On the other hand, FEA predicted lives 7, 1.3, 0.7 for group B crowns made of LD. Table 2 lists the maximum shear at the dentin-adhesive interface [MPa]. The maximum shear at the dentin-adhesive interface under the load of 150 N for group A made of zirconia is 5.5 MPa, while it is 5.3 MPa for the same group made of LD.

**Table 2.** Shear stress at the dentin-adhesive interface [MPa].

	Zirconia Cercon (ZC)			IPS e.max CAD (LD)		
Load (N)	Group A	Group B	Group C	Group A	Group B	Group C
75	4.1	3.8	3.6	4.1	3.7	3.5
120	4.9	4.5	4.1	4.8	4.5	3.7
150	5.5	5.1	4.7	5.3	4.9	4.1

## 4. Discussion

Clinically, all-ceramic restorations have failed due to the fatigue load generated mainly by the chewing process (Pieger et al. 2014; Fasbinder et al. 2010; Pogoncheff and Duff 2010). Fatigue failure is due to crack growth resulting from masticatory stresses. The life of the restorations depends on the thickness of the crown materials. Utilizing a thin crown prevents excessive removal of tooth structure which causes potential damage to the dental pulp or reduces the stability of the remaining tooth substance (Lameira et al. 2015). The aim of this study was to test the hypotheses that a thin crown made of CAD/CAM ceramic materials can withstand cyclic masticatory forces. FEM was used to test this hypothesis.

This study evaluated the lifetime of thin all -ceramic crowns under cyclic forces using both FEM and S – N fatigue behavior. Combining the S – N fatigue behavior with FEA is the proper approach and its results have great value in clinical practice. It is worth mentioning that only a few studies have used this approach (Homaei et al. 2016). Most studies have only performed static analysis. This could be the main reason why there is a gap between in vitro performance of dental materials and clinical observations. The FEM takes into account ceramic, dentin, adhesive, pulp. The method used in this study has great value because it has been validated and compared against previously published data. It is worth mentioning that the literature lacks information describing the mechanical behavior of the contact between different parts of the dental restoration.

Based on FEA results, the effect of the thickness of the crown on the in-vitro survival and fracture resistance of CAD/CAM all-ceramic material crowns was evaluated. Two types of cyclic mechanical loadings are applied. The range of mechanical loading values chosen is comparable to other in vitro studies (Abouelleil1 et al. 2015; Nihioka et al. 2018), simulating 5 years of intraoral use (Rosentritt et al. 2008, 2009). Although occlusal loads, chewing behavior, oral environment depend on the individual, a standardized condition is used to help simplify conditions and simulate clinically observed failures. Models that consist of over 320,000 elements were used for the entire simulations to guarantee that the results were mesh-independent.

The stress contours presented show that there are three points of contact. For the particular spherical indenter on three-cusp loading configuration, FEA predicts that the critical regions (highly stressed regions) are located slightly off the symmetry fissure plane between cusps. The common failure of ceramic crowns occurred in the contact area or central fossa. FEA successfully predicted the failure region which is the area of maximum tensile stress located in the central fossa of the occlusal surface (Dal Piva et al. 2018). The common failure of ceramic crowns occurred in the contact area or central fossa. ANSYS software has the capability to estimate the fatigue life. It is worth mentioning according to the failure theory of brittle material, the maximum tensile stress is responsible for the failure (Budynas et al. 2011). The maximum principal stresses are 43MPa, 82 MPa for group B zirconia which occurred under the buccal cusp under the axial load of 50 N and 150 N, respectively.

Group A crowns made of zirconia have lived less than two clinical years under cyclic fatigue axial load of 120 N. The fatigue life for group A crowns made of LD under the cyclic load of 120 N is shorter than 1 year. However, they lived longer than 5 years under the fatigue load of 50 N. To accurately simulate the chewing process, the second type of cyclic loading is applied (axial compression followed by .0 8 mm sliding in the direction of buccal). Similar behavior is observed under the action of the combined loading. Group B crowns made of Zirconia have lived longer than three clinical years under the action of 120 N axial load followed by 0.8 mm sliding motion. On the other hand, group A and B crowns made of LD survived less than a year under the same type of loading. These findings were inconsistent with previous vivo study (Fasbinder et al. 2010; Pieger et al. 2014). It is worth mentioning that group C crowns made of either zirconia or LD have lived longer than five clinical years under loads less than 75 N. This is in line with a clinical investigation that found lithium disilicate crowns worked and that the material was a good option for all-ceramic crowns (Fasbinder et al. 2010; Pogoncheff and Duff 2010). The factors of safety guarding against fatigue failure for group C zirconia crowns have large values which imply that the thick crown preparation is not necessary. FEA predicted that zirconia crowns can be safely made thin and still has sufficient fatigue resistance. Moreover, the effect of applying sliding motion has reduced the expected life of the can be safely crown materials. The limitations of this analysis could be summarized as follows; a) FEM analysis did not consider the actual behavior of the cement, which is essential for the survival and resistance of all-ceramic materials, b) deterioration of low temperatures, saliva flow rate, and the impact of variations in pH were not taken into account. Future study should focus on investigating the effect of low-temperature degradation and the existence of acidic on the long-term survival rates.

## 5. Conclusions

The minimum thickness for all- ceramic crowns survived under fatigue loading was estimated using FEA. The three zirconia groups survived longer than 5 years under fatigues load less than 100 N. Crowns made of zirconia have more than 100% safety factor against fatigue failure, even under the highest intraoral stress levels. The fatigue life for the ZC and LD crowns with a thickness higher than 1.5 mm exceeded 5 years under an axial load of 80 N. The fatigue strength of zirconia showed superior performance compared to LD. Ultra – thin crown (0.7 mm) made of LD; under load 50 N, has survived longer than 5, respectively. On the other hand, their life was shorter than 5 years under the load of 75 N. The predicted life and the failure mode show good agreement with experimental data and clinical observation. For all models studied, the maximum principal stresses are located at the contact area and central fossa of the occlusal surface. Finally, 50 μm thickness of dual-cure resin cement can withstand the shear stresses developed under the chewing process.
